# Factors influencing participation dynamics in research for development interventions with multi-stakeholder platforms: A metric approach to studying stakeholder participation

**DOI:** 10.1371/journal.pone.0223044

**Published:** 2019-11-14

**Authors:** Murat Sartas, Piet van Asten, Marc Schut, Mariette McCampbell, Moureen Awori, Perez Muchunguzi, Moses Tenywa, Sylvia Namazzi, Ana Sole Amat, Graham Thiele, Claudio Proietti, Andre Devaux, Cees Leeuwis

**Affiliations:** 1 Knowledge, Technology and Innovation Group, Wageningen University, Wageningen, The Netherlands; 2 International Institute of Tropical Agriculture, Kigali, Rwanda; 3 International Institute of Tropical Agriculture, Kampala, Uganda; 4 Makerere University, Kampala, Uganda; 5 International Vegetable Center, Kampala, Uganda; 6 International Potato Center, Lima, Perú; 7 International Potato Center, Quito, Ecuador; Ministry of Health and Sports, MYANMAR

## Abstract

Multi-stakeholder platforms have become mainstream in projects, programmes and policy interventions aiming to improve innovation and livelihoods systems, i.e. research for development interventions in low- and middle-income contexts. However, the evidence for multi-stakeholder platforms’ contribution to the performance of research for development interventions and their added value is not compelling. This paper focuses on stakeholder participation as one of the channels for multi-stakeholder platforms’ contribution to the performance of research for development interventions, i.e. stakeholder participation. It uses a quantitative approach and utilizes descriptive statistics and ARIMA models. It shows that, in three Ugandan multi-stakeholder platform cases studied, participation increased both in nominal and in unique terms. Moreover, participation was rather cyclical and fluctuated during the implementation of the research for development interventions. The study also shows that, in addition to locational and intervention factors such as type of the area along a rural–urban gradient targeted by the intervention and human resources provided for multi-stakeholder platform implementation, temporal elements such as phases of research for development intervention objectives and the innovation development process play significant roles in influencing participation. The study concludes that contribution of multi-stakeholder platforms to the performance of research for development projects, programs, policies and other initiatives is constrained by locational and temporal context and conditional on the participation requirements of the objectives pursued by research for development intervention.

## 1. Introduction

Multi-stakeholder platforms (MSPs) have been attracting increasing attention from different agencies aiming to improve innovation and livelihood systems (hereafter systems). MSPs have been utilized to facilitate different research for development interventions [R4Ds] such as government policies [1] and international research and development programmes and projects [2]. The literature on MSPs in livelihoods research covers a broad range of dimensions, including agriculture [3], natural resource management [4,5], environment management [6] and health [7]. Specific forms of MSPs include, among others, innovation platforms [3], public private partnerships [8,9], sustainability platforms [10] and learning alliances [11].

R4Ds aim to improve complex systems and require approaches and tools that enable holistic identification and analyses of both constraints and opportunities [12,13]. This implies that R4Ds need to respond to the needs and challenges faced by different stakeholder groups [14–16], making collective action and multidisciplinary, multi-stakeholder partnerships between research and development actors key for their performance [7,13,17]. MSPs have gained momentum in the R4D world, notably because of their ability to foster collective action across multi-disciplinary actors [6,7,18]. Their popularity is especially high in low- and middle-income countries where partially or non-participatory approaches have frequently been reported as insufficient to improve systems [17].

Although MSPs are popular, available evidence on their specific contribution to the performance and impact of R4Ds [hereafter MSPs’ contribution] has been scarce. MSPs have been reported to contribute to achieving objectives of R4Ds [7,19,20], but also to ‘failures’ [1,4,21,22]. Consequently, several studies have tried to identify the factors that influence MSPs’ contribution. Many of these studies converge around the role of several processes that influence MSPs’ contribution i.e. process drivers [23].

Among these process drivers, stakeholder participation [hereafter participation] has often been identified as a key indicator [4,24,25]. Stakeholders’ interest and participation in R4D events are considered to be related to the success of R4Ds [26,27]. In addition, MSPs are assumed to align participation to current activities of R4Ds [24,25]. In other words, in their ability to enhance higher and more compatible participation in R4Ds [28], MSPs are considered to increase the performance of R4Ds. Therefore, better understanding of participation in R4Ds when it is organized through MSPs has the potential to contribute to increasing the performance of R4Ds and scaling R4D innovations in low- and middle-income intervention contexts.

This study aims to contribute the literature by providing scarce quantitative evidence about MSPs’ contribution using descriptive statistics and time series approach. Existing studies on participation that uses a time series approach do not explicitly refer to MSPs and either have very limited in scope [29,30] or uses participation as an explanatory or independent factor rather than a dependent variable [31–36]. This study is one of the first studies that focuses on understanding participation dynamics and various factors contributing to these dynamics within R4Ds and investigates participation as the dependent variable.

## 2. The conceptual framework and methodology

### 2.1 R4D interventions and MSPs

The R4D literature has various definitions for R4Ds and MSPs. Based on a review we made for this study, we differentiate R4Ds with three major characteristics. First, systematic research plays a vital role, which is reflected on the allocation of an important share of R4D resources on systematic research [37,38]. Second, R4Ds utilize a systems approach in which multiple objectives and innovations are pursued and the interactions among and between objectives and innovations are explicitly articulated [37,39,40]. Third, R4Ds include participatory approaches [3,38,39,41] and multi-disciplinary teams [38,40,42,43].

Multi-stakeholder platforms [MSP] are defined as decision-making bodies [44] or round-tables where a diversity of stakeholders [5] get together to get things done [45]. This description is comprehensive and different approaches such as public private partnerships [8,9,46–49], innovation platforms [3,50–53] and sustainability platforms [10,54,55] are considered as MSPs. We characterize MSPs with several traits. First, they are not a single event but a series of interconnected events. Second, both research and non-research stakeholders are involved in and influence intervention decisions on different activities such as analysis of problems, design and implementation of research plan.

Therefore, R4D interventions organized though MSPs present a specific approach to implementation of system interventions and MSPs is a specific form of stakeholder involvement. Although, every R4D carries participatory elements, not all R4Ds have an MSP. In the R4D literature, there are various examples of interventions which are both R4Ds and MSPs, either R4D or MSP, or none [Table 1]. In this study, we focus on the intervention group which are both R4Ds and MSPs.

**Table 1:** Examples of interventions which carry different R4D and MSP characteristics in different R4D fields.

	Dimension	With MSP	Without MSP
**R4D Interventions**	Agriculture	Adekunle & Fatunbi, [56], Schut et al. [3]	Beers & Geerling-Eiff [1], Giuliani [57]
	Environment	Bäckstrand [58], Derak et al. [59]	Reed [60], Hermans et al. [61]
	Natural Resource Management	Hämäläinen et al. [62], Warner [5]	Prell et al., 2009 [63], Walker et al., 2010 [64]
	Health	McHugh et al. [7], Kasonde and Campbell [65]	Delisle et al. [37], Whitworth [66]
	Other	Barlow et al. [67], Bebbington & Farrington [68]	Reypen et al. [8], Roloff [69]
**Non-R4D Development Interventions**	Agriculture	De Zeeuw [70], Fleury et al. [71]	Thompson et al. [72], Pretty et al. [73]
	Environment	Abbott [46], Bosher et al. [74]	Meyer et al. [75], Saysel et al. [76]
	Natural Resource Management	Warner [77], Fliervoet et al. [78]	Steinmann et al. [44], Agarwal [79]
	Health	Magesa et al. [80], Eggersdorfer & Bird [47]	Yasuoka & Levins [81], Berti et al. [82]
	Other	Huang et al. [83], Mayangsari & Novani [84]	Beall & Todes [85], Balan et al. [86]

### 2.2 Definition of participation as a driver of R4D intervention performance

The relation between participation and using an MSP approach in R4Ds has been the subject of many studies [4,5,24,56]. However, the understanding of what participation means varies significantly [87]. For instance, participation has been defined as [1] simple attendance at R4Ds events [88], [2] continuous involvement in these events [89,90] or active influence on R4Ds’ agendas [60,88] etc. Different types of participation include passive participation, participation by consultation, functional participation, empowering participation and interactive participation [28,88].

In this paper, we use a simple definition of participation as referring to stakeholders’ physical attendance in diverse R4D events since it is the most common denominator of all the participation definitions we come across in the literature. Moreover, physical attendance is a prerequisite for more comprehensive definitions of participation. We are aware that studying participation in this definition might not be sufficient to cover diversity of definitions available in the literature, however such a definition is necessary part for all participation of definitions and inform studies on participation regardless of the specific definition they use. In this study, we further unpack participation and investigate it both in nominal terms–number of stakeholders–and in unique terms–number of distinct stakeholders. In addition, we investigate both non-cumulative and cumulative participation.

### 2.3 How does participation change?

Many studies have argued that participation changes when it is organized through MSPs [24,27,56,91], and the change is mostly in the form of an increase [26,27,92]. In addition, numerous studies have argued that, in R4Ds, MSPs go through several phases, whose details are discussed in section 2.4.3 [1,91,93–95]. These studies argue that participation increases as R4Ds advance through these phases.

### 2.4 Factors affecting participation

In the R4D literature, participation has been argued to depend on diverse factors. We classify them as [1] locational [96–98], [2] intervention-related [3,28,98] and [3] temporal [4,99–101] factors. In this paper, we study these three types of factors.

Among various factors in these three categories, we study seven specific factors in total, which are frequently discussed in the literature and that can be empirically investigated within the limitations of the resources provided for the study. We provide a list of other factors in supporting information [S1 File] to facilitate further research on the participation. One of the specific factors is locational [geographical location in which the R4D operates], three are intervention-related [funding, human resources provided by the R4Ds and type of event] and three are temporal factors [periods based on different R4D objectives, innovation development process and other time aspects].

#### 2.4.1 Location-related factors

The R4D literature includes many studies focusing on the location-related factors that influence stakeholder participation in R4Ds [93,97,102]. Comby et al. [93] argued that local communities’ interests were different from those of national and regional actors, and that consequently their participation in R4Ds is higher as these have a more direct impact on their livelihoods. Moreover, local media more frequently refer to specific local problems, attracting more widespread attention and interest in such issues. Raford [102] added that local actors’ deeper local expertise can increase the relevance of local actors for the R4Ds, and thus their participation. These arguments propose that stakeholder participation in R4Ds is higher at local level. In this paper, we investigate whether this proposition is valid in our cases to better understand the nature of participation and articulate the implications of using an MSP approach in R4Ds.

#### 2.4.2 Intervention-related factors

As with the locational-related factors, the literature includes multiple studies focusing on the intervention-related factors influencing participation. Examples include detail level of planning, i.e. clarity of impact pathways and theory of change [96], flexibility in implementation [98] and the type of organization managing the R4Ds project and/or MSP [3]. Among the intervention-related factors, three major aspects are more commonly instanced: [1] funding [4,28,96], [2] human resources [96,101,103,104] and [3] type of event [26,105].

It has been argued that the amount of funding provided to the MSPs increases participation by providing monetary incentives and improving the quality of MSP processes. In addition, allocated human resources have been argued to influence participation. The existence and quality aspects of champions who promote and improve the legitimacy of the MSPs [106–109], facilitators who manage communication and negotiation in the MSPs [16], organizers who arrange logistics and infrastructure [19], and documentation people who monitor and communicate R4D events, decisions and successes [3,16,19] have been especially argued to play important roles. Moreover, the type of R4D event is argued to influence participation. For instance, an implementation event that focuses on administrative and project management issues [e.g. financial planning meeting] typically includes few stakeholders, most of whom are employed by the organization managing the R4Ds, whereas a promotional event [product demonstration event] that aims to disseminate knowledge and innovations often includes many stakeholders.

#### 2.4.3 Temporal factors

The R4D literature discusses two major temporal factors that influence participation in R4Ds when MSPs are used. The first temporal factor is calendar-based periods. It is argued that participation increases or decreases in specific time periods. For instance, participation is argued to decrease in holiday periods [110,111] and increase in planting periods in R4Ds focusing on agriculture [112]. Consequently, the first factor we consider in the study is specific calendar-based periods, such as cultural events [e.g. festive seasons] and agricultural seasons. We will investigate several of such periods.

The second temporal factor is based on the time period R4Ds are implemented. Participation was discussed to increase as R4D implementation gets older [21,113, 114]. Our review on the temporal factors indicated two major specific commonly discussed ways of defining time periods, i.e. phasing. The first phasing is based on the changes in the R4Ds’ objectives and innovations. R4Ds have multiple objectives–such as improving productivity, decreasing malaria incidence–and work on different innovations that can serve these objectives, such as developing new higher yield seed varieties or new mosquito traps. The stakeholder configurations that best fit these objectives and innovations are different, and a change in these also causes change in participation. As R4Ds focus on specific objectives and innovations for a period [hereafter R4D objective phases] these periods influence participation.

The second phasing is based on the type of work done on a single innovation. In R4Ds, development of innovations goes through different periods. In general, initially, several potential innovations that can serve R4D objectives are discussed and prioritized. Later, R4Ds work on the prioritized innovations and improve them until they are disseminated and marketed to the stakeholders who can use them to improve their livelihoods. As these periods require the participation of different stakeholders to achieve different goals, a change in the periods influences stakeholder participation. In other words, the periods [hereafter, innovation development phases] influence participation.

In our review of temporal factors, we came across standard ways of capturing calendar-based periods and used a few of them. However, for periods of R4D objectives and innovation development phases, there are different arguments about how to define the phases. Therefore, we rapidly synthesized multiple literatures and used the conceptualizations articulated in the following paragraphs.

R4Ds aim to achieve multiple livelihood objectives such as improving food security, nutrition and health status of smallholder farmers, natural resource management, empowering women and youth. As targeting these objectives will typically require more resources than they have at their disposal, R4Ds often identify specific entry points to focus their activities. In addition, each of these objectives can be achieved through different innovations, leading to a process of prioritization and focusing.

The R4D literature suggests several ways to phasing for the time following the prioritization and focusing [27,69,93,94]. For this study, we synthesized two ways of phasing using the literature, i.e. R4D objectives and innovation development to study this time period. For the phases based on R4D objectives, we suggest a four-phase approach [Table 2]. The phases start with [1] an entry phase, when stakeholders prioritize and select the objectives and innovations on which the R4Ds will initially focus [56,99], followed by [2] a vertical progress phase, when stakeholders generate deeper understanding and ‘improve’ selected innovations [115], [3] a horizontal progress phase, when stakeholders focus on improving complementary innovations that are typically within the same value chain [103], or [4] a system progress phase, when stakeholders focus on improving the contribution of selected innovations to other objectives [67,116]. Depending on the MSP participants’ preferences, R4Ds might focus on vertical, horizontal or system progress.

**Table 2:** R4D objective based phases when participation is organized through MSPs.

Phase	Focus Objective	Focus Innovation	Description
**Entry**	N.A.	N.A.	Stakeholders discuss and prioritize objectives on which to focus in the R4D activities [i.e. focus objective], reflect on potential innovations that contribute best to the objective and prioritize critical innovation to be focused [i.e. focus innovation]
**Vertical progress**^**a**^	Entry objective	Entry innovations	Stakeholders apply, refine and improve focus innovation prioritized in the entry phase
**Horizontal progress**^**b**^	Entry objective	Complementary innovations	Stakeholders identify and work on innovations that are complementary to the focus innovation for achieving selected objectives
**System progress**	Different objective	Entry and complementary innovations	Stakeholders work on improving the same innovations [focus, complementary], contributing to other intervention objectives

a As the process of applying, refining and improving focus innovation requires *deeper* knowledge and experience about the focus innovation, we refer to this process as *vertical progress*.

b As complementary innovations are usually in the same value chains and work on these innovations crowds out the resources that can be used for vertical progress, we use *horizontal progress* to refer to the work on complementary innovations.

For the phases based on innovation development literature suggested several options [1,91,95,117]. Whereas Markard and Truffer [118] identified multiple phases ranging from formation to market growth, Edquist [91] proposed three phases: innovation generation, diffusion and use. In policy interventions, a four-phase approach including generation of a promising innovation, showing a business case for it, adoption/adaption by first movers and widespread adoption has been proposed [1]. The stepwise technology development assumption suggested that an innovation process starts with an idea, continues with basic research and technology formulation, followed by applied research, prototyping and demonstration, and ends with early and full commercial application [117]. As these phases require different stakeholder capacities, their existence will imply different stakeholder configurations and different sizes of participation during R4Ds [25,28]. In brief, these phases of innovation development might affect participation. In this paper, we utilize the three phases suggested by Edquist [i.e. generation, diffusion and use] because of their simplicity and generic character. To better accommodate innovation development in R4Ds, we add an initial phase in which the MSP goes through a participatory process of prioritization of innovations, resulting in the four phases described in Table 3.

**Table 3:** Innovation development phases in R4D.

Phase	Description	Typical Activities
**Innovation prioritization**	MSP stakeholders compare different innovations that will best fit the current objectives of the intervention and prioritize specific innovations to work on	Listing of innovation options, consulting about the options, collective prioritization
**Innovation generation**	MSP stakeholders design methods and implement practices in generating an innovation from scratch or from customization of intrinsic characteristics of an existing innovation to the geographical and institutional specifics of the location targeted by the intervention	Development of field protocols, field research, monitoring the results, validation of the models, prototypes
**Innovation diffusion**	Generated innovation is further discussed with innovation actors outside the MSP in the broader innovation system	Workshop with public sector representatives, meetings with technical organizations
**Innovation use**	The awareness and capacities of innovation end users, such as farmers, private sector organizations, are targeted for increased use of innovation in livelihood systems	Farmer and business fairs, community information campaigns, provision of training, publication of dissemination materials

### 2.5 Methodological framework

#### 2.5.1 Study context and study sites

We studied three MSPs in Uganda that were implemented under the CGIAR Research Programme on Integrated Systems for the Humid Tropics [Humidtropics], between 2013 and end 2016. The programme covered four regions across the globe, one of which was East and Central Africa, including Uganda. Humidtropics aimed to improve livelihood systems by reducing rural poverty, increasing food security, improving nutrition and health, and strengthening the sustainable management of natural resources. To reach these targets, the programme focused on improving agricultural productivity, access to affordable food and consumption of nutritious foods, decreasing environmental harm from agriculture, and improving the innovation capacity of the local innovation systems [107]. The programme aimed to optimize these outcomes to improve smallholder farmers’ livelihoods.

**Fig 1:**
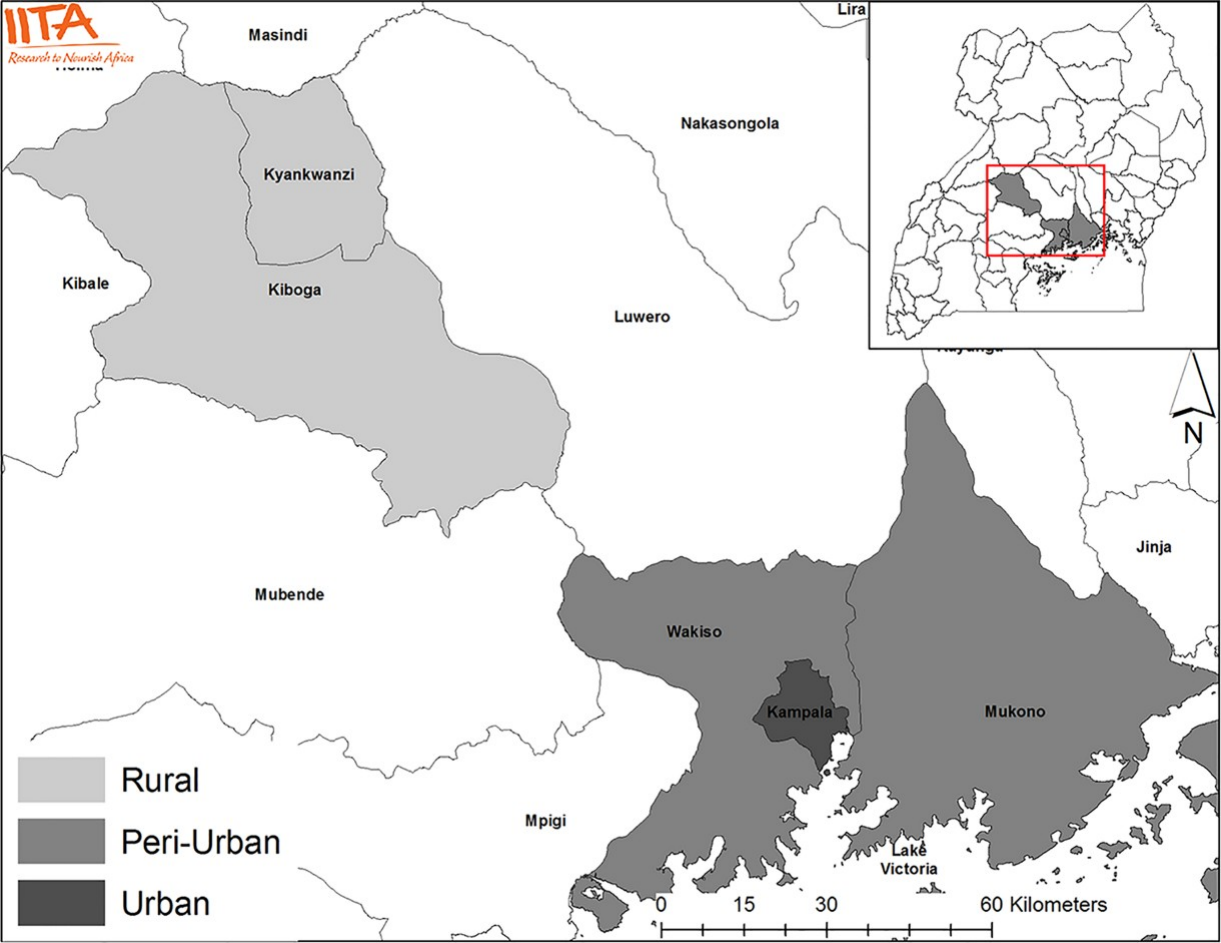
Map of Uganda indicating the locations of the three studied MSPs.

The Humidtropics programme in Uganda was organized through three MSPs. One of them was organized in Kampala–the economic and cultural capital of Uganda–another one in Kiboga and Kyankwanzi Districts, 123 and 155 km northwest of Kampala respectively, and the third one in Wakiso and Mukono Districts, 16 and 24 km east of Kampala respectively [Fig 1]. The programme included a diverse set of events, including meetings, field visits, experiments, capacity-building workshops and promotion events. The implementation and management of the events were supported by teams hosted within the organizations managing Humidtropics. The events in three sites kicked off in August 2013 with a big event, after which they were officially established in February 2014. The implementation and management teams included [1] champions–influential people who were financially and conceptually supportive of the MSPs in the organization leading the programme in Uganda–and district opinion leaders, [2] facilitators, both at national and at district scale, [3] organization and monitoring staff employed by the intervention working across the programme and their local assistants.

#### 2.5.2 Data collection

Data for the study were collected throughout the period 2013 to 2016, using two short surveys from the Learning System for Agricultural Research for Development [LESARD] [19]. The first survey was a LESARD Participant Profile, which recorded the characteristics of individuals participating in the R4D events [S2 File]. The participant profile was administered to all stakeholders who participated more than once in the events. The questions included the stakeholders’ organizations, their professional background, expertise subjects, scales of operation and role in the value chain group. The second survey was a LESARD Event Log, which recorded information about all the events in the R4D intervention [S3 File]. This included formal MSP meetings, but also smaller informal meetings where a subset of stakeholders met to pursue specific tasks agreed upon by the MSPs, field visits, workshops and promotional events. It covered location, start and end dates, event type and organizations funding different expenses of the events, as well as the information tools and documentation applied to the event. For the variables related to event types, minutes and audio and video materials were used to validate the content of the event.

#### 2.5.3 Data analysis

Data analysis was organized in two ways, namely, descriptive statistics and linear [time-series] regression. Descriptive statistics were used to present changes in participation in nominal and in unique terms throughout the R4D intervention. Linear time-series regression was done using ARIMA [119] in SPSS v.23. Specifically, we used IBM SPSS Time Series Modeller Procedure. The procedure uses partial autocorrelation functions, identify autoregressive orders [p], differencing [d], moving average orders [q] and outliers and transforms independent variables automatically [120] and shown to be a very good substitute for manual modelling [121]. Significant model coefficients are reported in Section 3. Analysis of participatory observations provided additional insights that enabled interpretation and discussion of our results.

Our study included two different sets of variables, namely, participation and factors affecting participation [Table 4]. Participation was measured by counting the number of stakeholders at an event. To study factors affecting participation, we focused on seven major factors, i.e. [1] geographical location, [2] funding, [3] human resources for the management and implementation of the MSP, [4] MSP events, [5] the phases of R4D development, [6] the phases of the innovation development process and [7] other time aspects. Each of these factors was investigated by analysing relevant variables in Table 4.

**Table 4:** Factors affecting participation.

Factors	Variables	Variable Description	Values
**Locational factors**			
**Geographical location**	Rural/urban characteristics of the location	The locations in which the stakeholders operate	Categorical: Kiboga-Kyankwanzi[1], Mukono-Wakiso [2], Kampala [3]
**Intervention factors**			
**Funding**	Share of funding [overall]	Share of funding not provided by the intervention [co-funding by other actors] for different events as a whole	Numerical: Ratio
	Share of specific funding [specific]	Share of funding provided by the intervention for different expenditures including mobilization, transportation cost, food, daily allowance, event venue, facilitation	Numerical: Ratio for each type
**Human resources**	Individual participation of MSP staff	Attendance of each type of MSP Staff, i.e. champions, facilitators, organizers and monitors	Categorical: present [1], absent [0]
	Total number of MSP staff	Number of total MSP implementation and management staff members participating in the events, including champions, facilitators, organizers and monitors	Numerical: Integer
**Events**	Type of event [detailed]	Type of event, including intervention implementation meeting, platform meeting, platform subgroup meeting, reflection meeting, capacity building event, field setup, field monitoring, researcher meeting, promotion event, fundraising event, specific events organized by MSP members and preparation events, taking an integer value for each type	Categorical: Different integer for each different type
	Type of event [research and delivery]	Type of R4D event classified into three groups based on focus content of the event	Categorical: research event [1], delivery event [2], both research and delivery [3]
**Temporal factors**			
**R4D objective**	Phase of the event	Phase of periods based on different objective-innovation bundles, taking an integer value for each phase. When the content of the event includes more than one progress event, they are categorized as multiple-progress	Categorical: entry [1], vertical progress [2], horizontal progress [3], systems progress [4], multiple progress [5]
**Innovation development**	Phase of the event	Phase of the innovation development processes, covering prioritization, generation, diffusion and use	Categorical: prioritization [1], generation [2], diffusion [3], use [4], mixed events [5]
**Time**	Production seasons	Production seasons of the focus commodity of the MSP	Categorical: rainy [1], dry [0]
	Specific periods	Long non-working periods such as Christmas and Easter breaks, national holidays	Categorical: Special event [1], otherwise [0]

We calculated rural/urban characteristics of the location by checking the administrative classification of the location provided as a response to the location question in the LESARD Event Log. Share of funding in overall and in specific terms were calculated by checking the responses given to the fund provision question in the Event Log. Each cost item, i.e. mobilization, transportation cost, food, daily allowance, event venue and facilitation, was given equal weight. Type of event was identified using the response given to multiple selection event type questions in the Event Log. Phases of periods based on different objective-innovation bundles and innovation development process were identified using the responses given to the event type question and validated with controlling the minutes and pictures of the events. Other time periods, i.e. production seasons and special breaks, were identified by the stakeholders within the social and geographical specifications of the locations in which the MSP was active.

## 3. Results

### 3.1 Participation and changes in participation

Participation in 411 R4D events ranged between a monthly average of 4 and 51. It presented similar trends within five different periods during the course of the R4D. In order to present the data in a more informative manner we named these periods from A to E and described the averages in each period. The periods are formulated using the observed visual patterns in participation plots; they are not results of any statistical inquiry. Nevertheless, the periods inform our inquiry on the changes in the participation especially in terms of the patterns the change in participation present.

**Fig 2:**
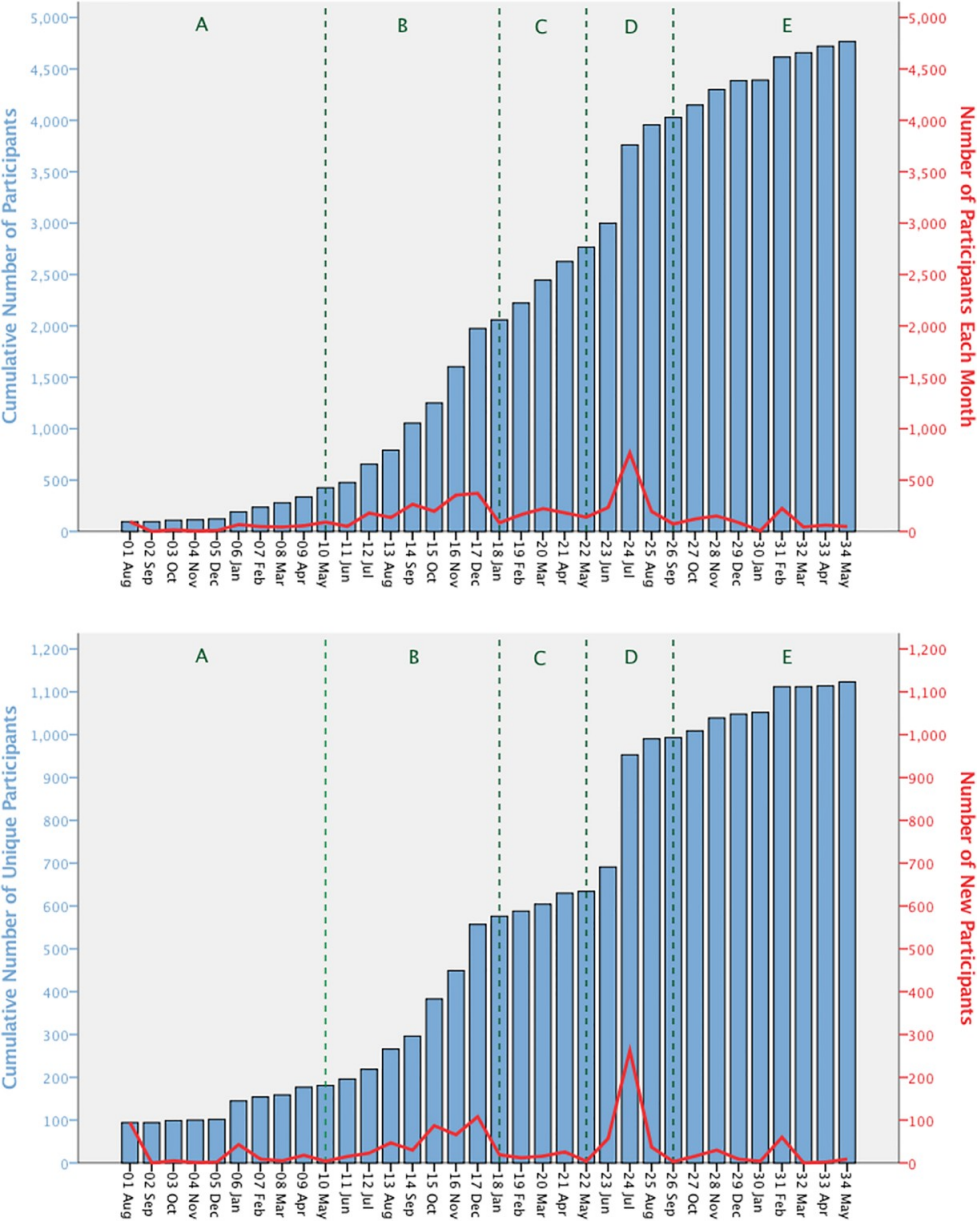
Number of nominal participants and unique participants in Uganda R4Ds. Nominal and unique participation in MSPs had similar patterns throughout the intervention period. Stable periods [A, C, E] were followed and preceded by periods of increase [B, D].

The average participation up to the tenth month after MSP establishment [Period A] was 31 per month [Fig 2]. Between months 11 and 18 [Period B] and between months 22 and 26 [Period D], there was an increase in average participation. In Period B, the average monthly participation was 173, and it was 288 in Period D. Between months 19 and 21 [Period C] and from month 27 onwards [Period E], average participation per month was lower than in the preceding months, with averages of 157 and 112.

During the three years of implementation, 4767 nominal and 1123 unique stakeholders participated in the R4D. Nominal and unique participation presented similar patterns across the MSP periods. During Periods A, C and E, participation in the MSPs was mostly stable, except during months 6 and 31 [Fig 2]. During Periods B and D, participation increased. In Period B, the increase accelerated continuously, whereas in Period E it peaked in month 24. In the first half of Period A, participation stagnated.

### 3.2 Factors affecting participation and changes over time

Location-related, intervention-related and temporal factors were significant in affecting participation [Table 5]. In terms of location-related factors, participation was relatively lower in urban areas, medium in peri-urban areas and higher in rural areas. Regarding the intervention-related factors, human resources, i.e. participation of facilitation, organization and monitoring staff in the R4D events, were significant. Higher investments in human resources were positively correlated with stakeholder participation. Among temporal factors, phases of R4D objectives and innovation development were significant. Participation was higher in later phases of both temporal factors [Table 5].

**Table 5:** Linear regression results for participation. Numbers in the third column represent the coefficients for statistically significant factors of participation with 0.01 [**] and 0.05 [*] confidence levels.

Factors	Variables	Model Coefficient	Description
**Locational factors**			
**Geographical location**	Location of event in rural–urban gradient	-3.37**	Participation is relatively low in urban locations, medium in peri-urban locations and high in rural locations
**Intervention factors**			
**Human resources**	Facilitator	2.11**	Participation is high at events when there are facilitation staff
	Organization and monitoring	1.19*	Participation is high at events when there are organization and monitoring staff
**Temporal factors**			
**R4D objectives**	R4D objective phase	4.45**	Participation is relatively low in entry phase events, medium in vertical progress, and higher in horizontal and system progress events
**Innovation development**	Innovation development phase	3.48**	Participation is relatively low in prioritization phase events, medium in generation events, and higher in diffusion and use events
**Model Information**			
**Time lag**	AR [1]	0.46**	
	R Square	0.36	
Only significant factors are included. The model uses ARIMA [1,0,18].			

Among the intervention-related factors, [1] share of funding provided for the events by the R4D project, [2] share of specific intervention funding to different expenditures including mobilization, transportation cost, food, daily allowance, event venue, facilitation, [3] type of event based on detailed event type, [4] type of event based on research and delivery and [5] participation of champions were insignificant. Among temporal factors, [1] production season and [2] other special periods such as long holidays were insignificant.

## 4. Analysis and discussion

### 4.1 Is participation dynamic?

Our study confirms that MSP participation is dynamic, as found also in previous studies [24,27,56,91]. The participation dynamics presented a cyclical pattern in the cases that we investigated. Periods of stagnation in the number of new stakeholders were followed by periods of expansion [Fig 2]. Participation in both nominal and unique terms was mostly stable in Periods A, C and E and changing in Periods in B and D. These changes in participation are partially caused by the combined effect of the significant factors discussed later in detail in section 4.2 that led to these dynamics aspects. As the explanatory power of the model is 36% [Table 5], other important factors beyond the scope of this study contributed to the patterns observed in these periods.

Our study showed that total participation increased both in nominal and in unique terms. More than 1100 unique stakeholders participated in the three MSPs [366 per MSP], totalling approximately 4750 nominal participations [Fig 2]. This confirms that both nominal and unique participation during R4Ds can increase when it is organized through MSPs [69,101] although it does not imply any direct causality between using the MSPs and increase in participation. As these numbers reflect the stakeholders that participated in two events or more, the outreach of R4Ds that apply the MSP approach when participation is organized through MSPs is likely to be higher than the numbers reported in this study. However, in non-cumulative terms, participation did not show a trend. In other words, average participation in a month was not higher or lower in the later period of the intervention in comparison to the earlier period.

### 4.2 How was participation influenced by location-related, intervention-related and temporal factors?

In the MSPs studied, different location-related, intervention-related and temporal factors influenced participation.

#### 4.2.1 Location-related factors

The investigated locational factor, geographical location along a rural–urban gradient, was significantly related to participation [Table 5]. Participation was highest in Kiboga-Kyankwanzi, medium in Mukono-Wakiso and lowest in Kampala. As Kiboga and Kyankwanzi are rural districts far from Kampala, and Mukono-Wakiso is a relatively close peri-urban area [Fig 1], we can argue that rurality is a relevant aspect that makes location significant. The decrease in participation along the rural–urban gradient confirms the locality argument. In other words, the higher interest of the local communities and the local media based in the rural areas might lead to higher participation in rural areas. The decrease in participation along the rural–urban gradient might be also due to the availability of alternative options for stakeholders. Stakeholders might have more opportunities and demands to allocate their time in the urban locations in comparison to peri-urban and rural locations where these opportunities are scarcer [122–124]. In addition, relative higher relevance [125] and urgency [126,127] of the R4D activities for the livelihoods of the residents of rural areas, in which agriculture is more relevant in relative terms, might lead to high prioritization of MSP events to other alternatives for allocation time.

#### 4.2.2 Intervention-related factors

Our study also showed that human resource allocation to manage and implement the MSP was significantly correlated to participation [Table 4]. Among the specific human resource contributions, the availability of facilitation, organization and monitoring was significant. Although statistical relations do not necessarily imply causality, the results support the commonly reported important role of facilitation for participation [1]. Although not frequently referred to in the literature, organization and monitoring are mentioned as important in many events. For instance, a field researcher in the Mukono-Wakiso platform in month 13 mentioned that ‘*Provisions of Mukono local administration for organizing of field and farmer visits helped to reach many farmers in different sub-counties*’.

Funding provided by the R4D intervention [in this case Humidtropics] to events as a share of the total cost of the events was not correlated with participation. Similarly, specific funding for mobilizing stakeholders to participate in the event, transportation of the stakeholders to and from the venue, provision of a daily allowance and food to participating stakeholders, using the event venue and facilitation of the event were not significantly correlated with participation when the influence of confounding factors is removed. This result does not confirm the arguments of some earlier studies [4,28,96] regarding the positive relation between funding and participation. It also indicates that monetary incentives might not be sufficient to increase participation, as opposed to the arguments in some of the literature [14,128].

One reason why funding is not significant could be that the amount of funding provided by the R4Ds is not sufficient to cover the opportunity costs of participation. MSPs consist of a continuous set of activities, and influencing the MSPs’ agenda might necessitate participation in the R4D events multiple times. When combined, participation in multiple events can create a large demand on stakeholders’ time. The time spent on the MSPs could be used on many other activities that would give stakeholders more direct benefit. Earlier studies have indicated that this may be particularly relevant for private sector stakeholders [25].

Type of event organized by the R4Ds through MSPs was not significantly correlated with participation. Participation was not significantly different between research and delivery events. In addition, participation varied for all 12 event types over the course of the R4D interventions. The insignificant result implies that geographical location where the event was organized was more relevant for participation than the type of event. The same type of event might attract different participation in rural and urban areas. This was indeed the case in our study. On average, both research and delivery events attracted more participation in rural areas [18.5 and 24.2 persons per event, respectively] than in urban areas [6.6 and 6.7 persons per event, respectively]. Similarly, in 9 of the 10 events organized both in rural and in urban areas, participation was higher in rural areas.

#### 4.2.3 Temporal factors

In our study, calendar-based periods were not significantly related to participation. Production season and specific time periods like the December festive season were insignificant. However, both R4D objective and innovation development phases were significant [Table 5]. This confirms findings from the literature in which changing MSP participation over time is related to phases of R4D objectives and innovation development [1,91,93] and validates that work content based phasing can explain the changes in participation in R4Ds.

Although agriculture was central in our empirical cases, high dependency of agriculture in climatic seasons was not enough for making calendar-based period significant. In addition, considering that influence of calendar based periods in other R4Ds focusing on natural resource management, health and environmental dimensions of livelihoods would be less than agricultural dimensions presenting strong seasonality [129,130], it can be argued that MSPs can make participation in R4Ds more continuous.

## 5. Conclusion

Our study has shown that MSPs are conducive to increasing the cumulative number of stakeholders participating in the events of R4Ds. However, the increase is not valid for average terms. Moreover, locational and temporal factors might have a higher influence than intervention factors, implying that participation is more autonomous in nature than induced or influenced by the R4D intervention. Therefore, we conclude that MSPs’ contribution to the performance of R4Ds in increasing participation is constrained by “locational and temporal context” and conditional, depending on the duration of the participation necessary to achieve the R4D objectives. In other words, MSPs might not increase participation significantly in some locations and in some periods and R4Ds might not have the ability to increase participation in those places and times.

The findings in our study imply that different stakeholders can participate throughout the course of the R4D. However, MSPs are not effective in increasing average participation. Thus, if the R4D objective requires continued participation by a high number of stakeholders, such as enhancing collective action or supervising R4D events, MSPs’ contribution is limited. In addition, our study has shown that participation fluctuates or is cyclical. Therefore, the potential contribution of MSPs to R4Ds changes over time. Consequently, monitoring participation and scheduling R4D events based on participation might be necessary to make best use of the MSP approach.

We conclude that since the contribution of MSPs to the participation of stakeholders in R4D interventions are constrained, conditioned and limited, their use need to be contemplated critically, especially by R4Ds aiming transformative changes in different dimensions of livelihoods, e.g. agriculture, health, environment and natural resource management that require intense participation of diverse stakeholders. Consequently, other complementary tools and approaches need to be used strategically and tactically to increase participation in R4D projects, programs, policies and other initiatives.

In this study, we aimed to provide one of the early systematic quantitative evidence on the contribution of MSPs to the participation in R4D intervention and therefore used a metric approach. Since a metric approach requires reduction of the detailed information to identify and test standard measures, we could only partially complement our results using the rich qualitative data we accumulated. We intend to use this rich qualitative evidence to achieve a more complete picture and further improve the validity of the results in follow up research. Another potential study to improve the result of this study is to investigate broader aspects of participation and investigate how improving the complexity of the definition of participation influence the results. A third research endeavour that can improve the result of this study is to systematically explore the reasons why the participation followed the cyclical pattern presented by the results.

## Supporting information

S1 File Annex 1 List of Interventions. A list of interventions, domains they operate and factors referred in explaining participation in the interventions.
(DOCX)

S2 File Annex 2 LESARD participant profile Uganda. Characteristics of the participants are documented by using a short survey, LESARD Participant Profile.
(DOCX)

S3 File Annex 3 LESARD event log Uganda. Characteristics of the events were documented by using a short survey, LESARD Event Log.
(PDF)
