# Supplementary material for: Factors influencing participation dynamics in research for development interventions with multi-stakeholder platforms: A metric approach to studying stakeholder participation
Source: PLoS One. 2019 Nov 14;14(11):e0223044. doi: 10.1371/journal.pone.0223044 (PMC6855456; doi:10.1371/journal.pone.0223044)
Supplement: S1 File — A list of interventions, domains they operate and factors referred in explaining participation in the interventions. (DOCX) [file pone.0223044.s001.docx]

**Annex 1 : LESARD Participant Profile**

This form is applied to all individuals attending an event FIRST TIME in the whole process. If you filled this time before please inform the organizer.

# First name and Surname

1. **Which organisations do you represent?**

Please specify all the organisations you are affiliated and working currently.

# What are your professions?

Please specify all relevant. Some examples are farmers,agronomist, economist.

# What are your expertise subjects?

Please specify all relevant. Some examples are maize growing, rubber trade, innovation systems...

# Please choose the boxes appropriate for yourself

Please choose all three applicable options

*Check all that apply.*

Female Male Youth Adult Single Married

Other:

# To which stakeholder groups do you belong?

Multiple answers possible. Please specify all relevant.

*Check all that apply.*

Input suppliers (seed, fertilizer) Farmer, primary producer Trader or broker

Processor

Retailer or wholesaler Consumer groups

Extension officers/public local technical staff Other business

Farmers organisations

Local or national researchers International researchers Local NGO

Local politician Local media National Politicians National NGO National Media International NGO

Other:
