# Supplementary material for: Factors influencing participation dynamics in research for development interventions with multi-stakeholder platforms: A metric approach to studying stakeholder participation
Source: PLoS One. 2019 Nov 14;14(11):e0223044. doi: 10.1371/journal.pone.0223044 (PMC6855456; doi:10.1371/journal.pone.0223044)
Supplement: S2 File — Characteristics of the participants are documented by using a short survey, LESARD Participant Profile. (DOCX) [file pone.0223044.s002.docx]

**LESARD Event Log**

This form aims to collect information about the event organized as a part of multi-stakeholder platform process. It needs to be administered in ANY SIGNIFICANT EVENTS which will have an impact on the evolution and decision making in the multi-stakeholder process. It is advised to start with the form before the event and finalize it after the event is completed.

# Filled in by:

1. **Name of the event**

Please specify the name of the event, if there is any

# Start

Please specify date and time

*Example: December 15, 2012 11:03 AM*

# End

Please specify date and time

*Example: December 15, 2012 11:03 AM*

# Location

Please specify the event venue, section in the city and city

# Type of event

Please check all applicable. More than one answer is possible.

*Check all that apply.*

Preparation meeting for the upcoming event Platform meeting

Platform sub-group/team meeting Reflection meeting

Coordination or planning meeting between intervention actors Capacity creation or building / training

Field trial setup

Monitoring the field, data collection Research meeting for researchers

Promotion event of the platform and its activities Fundraising event for the platform

Specific events organized by platform member organization (s) Other:

# Which information tools are utilized for the event?

Please check all relevant. Multiple answers are possible. The information covers all information provided before and during the event.

*Check all that apply.*

No information about the event was provided to participants

E-mails about the events specifics were sent before the event Participants were called and were given update before the event

Participants were sent a calendar invitation and short description before the event Participants were send brochures, letters and other hardcopy materials before the event Hand outs are distributed during the event

Presentations made using power-point, Prezi etc. during the event. Organizers give oral updates during the event

Specific information tools and exercises done during the event

Results from the experiments and process learning discussed during the event Other:

# Which documentation is done for the event?

Please choose all relevant. Multiple answers are possible.

*Check all that apply.*

None

Meeting minutes Boards, blackboards Pictures

Event log

Dynamic learning agenda Other Questionnaires Audio record

Video record

Posters, papers, card created in the event E-mails, other online means

Reports about the event Other:

# Resource contribution except labor or own time

Please check the all appropriate boxes. Multiple answers are possible. Please specify the other actors if relevant

*Mark only one oval per row.*

Lunch/dinner

Coffee/Tea, Snacks and other drinks Transportation allowance/ reimbursement

Fixed per-diem

Costs of the event venue Facilitation costs

Other costs

Farmers CGIAR

Center

Local government

Local or

national NGO

National government

Intern. NGO

National research and academic system

Private sector

# Further observations/comments of the monitor

Please specify all remarks about the event that Humidtropics and the team need to notice
