# Supplementary material for: Factors influencing participation dynamics in research for development interventions with multi-stakeholder platforms: A metric approach to studying stakeholder participation
Source: PLoS One. 2019 Nov 14;14(11):e0223044. doi: 10.1371/journal.pone.0223044 (PMC6855456; doi:10.1371/journal.pone.0223044)
Supplement: S3 File — Characteristics of the events were documented by using a short survey, LESARD Event Log. (PDF) [file pone.0223044.s003.pdf]

No	Intervention	Is R4D?	Domain	With MSP?	Literature	Explicitly investigated factors of participation	Study Methodologies
1	United Nation Environmental Program (UNEP)	No	Environment	Yes	Abbott, 2012	Expectation of direct benefit, partnership opportunity with business sector,	Qualitative
2	Sub-Saharan Africa Challenge Program	Yes	Agriculture	Yes	Adekunle & Fatunbi, 2012	Expectation of direct benefit, a clear way to make contribution.	Qualitative, Descriptive Statistics
3	Community Forestry Groups	No	Natural resource	No	Agarwal, 2011	Rules of entry, social norms, social perceptions, entrenched territorial claims, personal endowments and attitudes, household endowments and attitudes	Qualitative, Descriptive Statistics
4	World Submit on Sustainable Development	Yes	Environment	Yes	Bäckstrand, 2006	An influential initiative	Qualitative
5	US and EU biofuel initiatives (Review)	No	Other (Biofuel)	No	Balan et al., 2013	None	Qualitative
6	UK Telecare Schemes	Yes	Other (Telecare)	Yes	Barlow et al., 2006	Careful project management, capable project manager	Qualitative
7	Cato Major (Urban development) Project	No	Other (Urban De	No	Beall & Todes, 2004	Sensitivity to social dynamics especially gender	Qualitative
8	Overseas Development Institute facilitated government NGO collaboration projects (Review)	Yes	Other	Yes	Bebbington & Farrington, 1993	Funding, influence sharing arrangements	Qualitative
9	Dutch Agricultural Policy	Yes	Agriculture	No	Beers & Geerling-Eiff, 2014	None	Qualitative
10	30 Agriculture interventions that measured impact on nutritional status (Review)	No	Health	No	Berti et al., 2004	None	Qualitative, Descriptive Statistics
11	Generic UK Flood Hazard Management Interventions	No	Environment	Yes	Bosher et al., 2009	None	Qualitative
12	Cities farming for the Future & From Seed to Table	No	Agriculture	Yes	De Zeeuw, 2010	Process facilitation, government incentives	Qualitative
13	The Global Forum for Health Research Funded Interventions (Review)	Yes	Health	No	Delisle et al., 2005	NGO facilitators, expectation of direct benefit, continuity of the dialogue, reciprocity of dialogue, existance of different participation modalities, funding, recognition of individual contribution, training	Qualitative
14	Ecological Restoration in Northern Morocco	Yes	Environment	Yes	Derak et al., 2006	Process facilitation, dependence on local resources	Qualitative and Quantitative
15	DSM-World Food Programme Partnership & Sustainable Evidence-Based Actions for Change & The National Complementary Feeding Program in Chile	No	Health	Yes	Eggersdorfer & Bird, 2016	NGO facilitators, transparency	Qualitative
16	Implementation of Sustainable Agriculture and Rural Development in Alpine Mountains (IMALP)	No	Agriculture	Yes	Fleury et al., 2008	Champions, recognition of individual contribution, size of the economic impact of the intervervention	Qualitative
17	Rijnwaardense Uiterwaarden (Dutch Floodplains)	No	Natural resource	Yes	Fliervoet et al., 2017	Willingness of the participations to collaborate, recognition of interdependence, feeling of responsibility, existance of common vision	Qualitative
18	Chile Valle de Colchagua cluster network	Yes	Agriculture	No	Guiliani, 2013	reciprocity	Quantitative
19	Water Level Management Policy for a Regulated Lake-river System	Yes	Natural resource	Yes	Hämäläinen et al., 2001	Compatibility of the visions, interactivity of thepl process,	Qualitative
20	5 Sustainability monitoring in different provinces in the Netherlands: Brabant, Zeeland, Limburg, Flevoland and Utrecht	Yes	Environment	No	Hermans et al., 2011	Indivodual influence on decisions, context, monitor	Qualitative
21	Multi-stakeholder Platform of a mining company Guizhou Province of China	No	Other (Mining)	Yes	Huang et al., 2017	Institutionalized process	Qualitative
22	Zambia Forum for Health Research	Yes	Health	Yes	Kasonde & Campbell, 2012	Selection of whom to invite	Qualitative
23	Multi-stakeholder Action for Taking Insecticide-treated Nets to National Scale (NATNETS)	No	Health	Yes	Magesa et al., 2005	None	Qualitative, Descriptive Statistics
24	Indonesia Bandung Smart City Project	No	Other (Urban De	Yes	Mayangsari & Novani, 2015	Regulations, clear vision for the objective of MSP	Qualitative
25	Aligning Forces for Quality Initiative (AF4Q)	Yes	Health	Yes	McHugh et al., 2016	None	Quantitative
26	Carbonization Technology Implementation Projects (Review)	No	Environment	No	Meyer et al., 2011	None	Quantitative
27	UK Peak District National Park Management Initiative	Yes	Natural resource	No	Prell et al., 2009	Reciprocity, emotional intensity	Quantitative
28	286 Agricultural Development Interventions (Review)	No	Agriculture	No	Pretty et al., 2006	None	Quantitative
29	Generic cases on social learning in environmental context	Yes	Environment	No	Reed, 2010	Facilitation	Qualitative
30	The European Medical Information Framework (EMIF)	Yes	Other (Manufactu	No	Reypen et al., 2016	Direct benefit, clear value objective	Qualitative
31	Fair Labor Network & United Nations Global Contact & Global Reporting Initiative & the World Commission on Dams & UK Banz stakeholder dialogue of the Puma AG & Grainger Town Project	Yes	Other	No	Roloff, 2008	Relevant experience, language, time, energy, travel funds, participation experience, maximum number of overall participants	Qualitative
32	Southeastern Anatolian Project (GAP)	No	Environment	No	Saysel et al., 2002	None	Quantitative
33	Integrated Systems for the Humidtropics Research Program	Yes	Agriculture	Yes	Schut et al., 2016	None	Qualitative
34	Generic Systematic Review identified interventions working on Schistosomiasis and water resources	No	Natural resource	No	Steinman et al., 2006	None	Quantitive
35	Natural Products Crop Protection Discovery Programme	No	Agriculture	No	Thompson et al., 2000	None	Quantitive
36	African Union's New Partnership for Africa's Development (NEPAD)	Yes	Natural resource	No	Walker et al., 2010	None	Qualitative

No	Intervention	Is R4D?	Domain	With MSP?	Literature	Explicitly investigated factors of participation	Study Methodologies
37	Multiple water resource management interventions	Yes	Natural resource	Yes	Warner, 2006	Clear mandate, maximum number of overall participants, facilitation, direct benefit, opportunity cost of participation, quick gains	Qualitative
38	Multiple water resource management interventions	No	Natural resource	Yes	Warner, 2007	Recognition of interdependence,	Qualitative
39	Multiple Initiatives on capacity strengthening of health research in sub-Saharan Africa	Yes	Health	No	Whitword, 2008	None	Qualitative
40	60 interventions on anopheline ecology and malaria incidence as a consequence of deforestation and agricultural development (Review)	No	Health	No	Yasuoka & Kevin's, 2007	None	Quantitative
